# The Genomic Characterization of Two Microbacterium foliorum–Specific Bacteriophages, QuadZero and AnnaLie

**DOI:** 10.1128/mra.00208-22

**Published:** 2022-06-06

**Authors:** Jennifer Cook-Easterwood, Donovan J. Bethel, Irene Kurikose, Joanna Katsanos

**Affiliations:** a Department of Biology, Queens University of Charlotte, Charlotte, NC, USA; DOE Joint Genome Institute

## Abstract

The microbacteriophages QuadZero and AnnaLie were isolated from soil samples from Charlotte, NC, and were classified into EA and EB clusters, respectively. QuadZero has a 40,140 base-pair double-stranded DNA genome with 62 predicted protein coding genes, whereas AnnaLie has a 41,665-bp genome with 71 predicted protein coding genes.

## ANNOUNCEMENT

We report the genome sequences of two microbacteriophage, QuadZero and AnnaLie, that were isolated from two different soil samples (35.189N and 80.831W and 35.264N and 81.015W, respectively) as part of the Howard Hughes Medical Institute’s (HHMI) Science Education Alliance-Phage Hunters Advancing Genomic and Evolutionary Science program (SEA-PHAGES) (1). These genome sequences contribute to the greater understanding of the diversity and abundance of microbacteriophage species. This information can provide a better understanding of their relationship to their bacterial host and ecological role (1).

**FIG 1:**
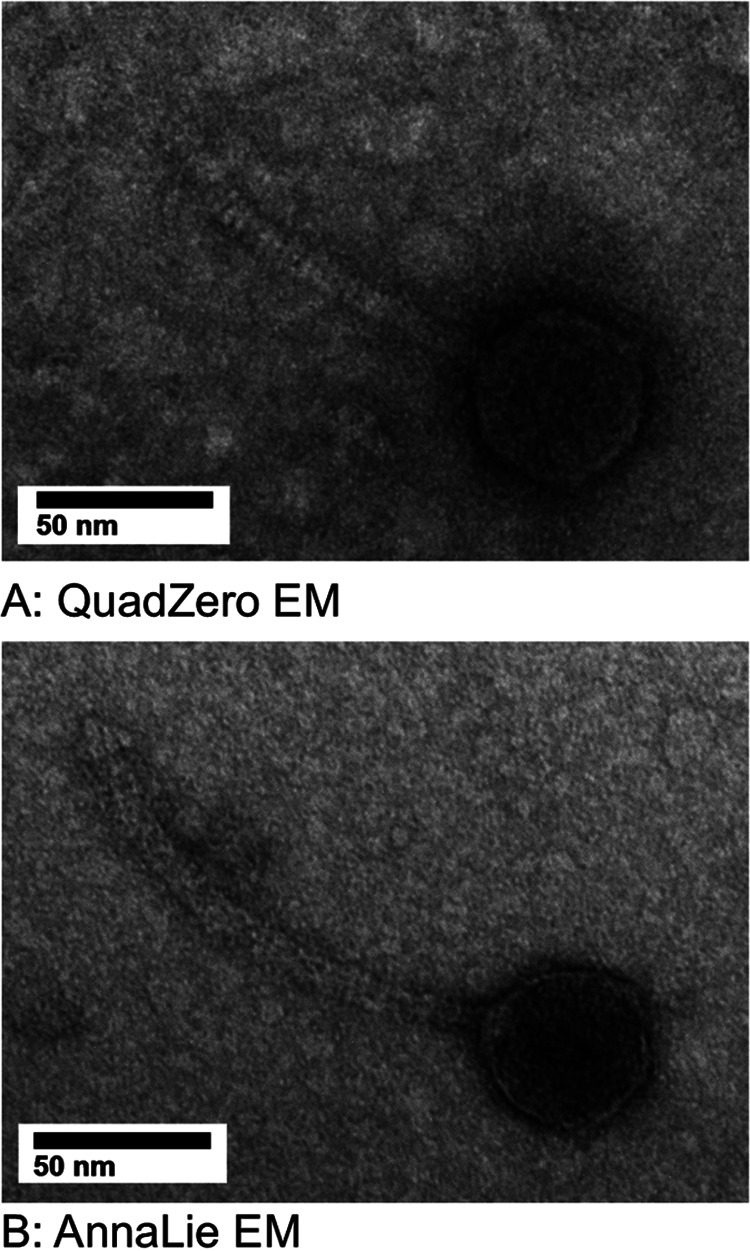
Transmission electron microscopy of the microbacteriophage Quadzero and AnnaLie. High-titer lysate was attached to a 300 copper mesh grid and negatively stained with 2% uranyl acetate. Imaging performed using a JOEL JEM-1400Plus Transmission Electron Microscope revealed siphovirus morphology for both phage. Quadzero had a capsid diameter of 50 nm and tail length of 113 nm (A), whereas AnnaLie had a capsid diameter of 47 nm and tail length of 113 nm (B).

Using the protocols provided in the HHMI’s SEA-PHAGES discovery guide, (https://seaphagesphagediscoveryguide.helpdocsonline.com/home) the microbacteriophage were isolated using the enriched isolation protocol and the bacterial host Microbacterium foliorum NRRL B-24224 (provided by the Pittsburgh Bacteriophage Institute at University of Pittsburgh) grown in PYCa liquid media at 30°C for 5 days. Isolation was followed by three to four rounds of plaque assays to purify the phage, followed by production of high-volume lysates to amplify the phage. Subsequent negative staining transmission electron microscopy revealed that QuadZero and AnnaLie have siphovirus morphology. A representative virion for QuadZero had a capsid diameter of 50 nm and tail length of 113 nm, while a representative virion for AnnaLie had a capsid diameter of 47 nm and a tail length of 141 nm (Fig. 1) as measured by imageJ v.1.53k (2).

Genomic DNA was isolated from the bacteriophage using the Promega Wizard DNA cleanup kit and prepared for sequencing with a NEB Ultra II DNA kit. Genomes were sequenced at the Pittsburgh Bacteriophage Institute with an Illumina MiSeq instrument, yielding 515,401 reads of 150-base single-end reads (QuadZero) and 813,568 reads of 150-base single-end reads (AnnaLie). The raw reads were assembled using Newbler 2.7 (3) with default settings in each case yielding a single contig for each phage. Consed version 29 (4) was used to check for genomic termini, accuracy, and completeness, as previously described (5). QuadZero had a genome length of 40,140 bp with a fold coverage of 1787, a G+C% content of 63.6%, and circularly permuted ends. AnnaLie had a genome length of 41,665 bp with a fold coverage of 842, a G+C% content of 66.6%, with 10-base single-stranded 3’ extensions (5′-TCTCCCGGCA−3′).

The protein coding regions of the phage genomes were predicted using Glimmer v3.02 (6) and GeneMark v2.5p (7) embedded within DNA Master v5.02 (http://cobamide2.bio.pitt.edu/computer.htm) using the settings described in HHMI’s SEA-PHAGES Bioinformatics Guide (https://seaphagesbioinformatics.helpdocsonline.com/home). Gene starts were predicted using Starterator (http://seaphages.org/software/), while protein function was determined using NCBI BLASTp 2.7 (8), Phamerator (9) and HHPred v3.0beta (10). QuadZero contained 62 predicted protein-coding regions with 24 putative functions, whereas AnnaLie contained 71 predicted protein-coding genes with 31 putative functions. No tRNA sequences were identified in either bacteriophage genome by Aragorn v1.1 (11) and tRNAscan-SE v 2.0.6 (12). QuadZero and AnnaLie both contained putative translational frameshifts in the tail assembly chaperone genes.

Clustal Omega alignment (https://www.ebi.ac.uk/Tools/msa/clustalo/) (13) using default settings indicated a 71% average nucleotide sequence identity (ANI) between QuadZero and Alakazam (GenBank accession number MT024862) and a 70.95% average nucleotide sequence identity (ANI) with Neferthena (GenBank accession number MH697589), both of which are members of the EA cluster, a microbacteriophage cluster. AnnaLie had a 94.86% average nucleotide sequence identity (ANI) with SansAfet (GenBank accession number MN329675), and a 90.22% average nucleotide sequence identity (ANI) with BubbaBear (GenBank accession number MK814753), both of which are members of the EB cluster, also a microbacteriophage cluster.

### Data availability.

GenBank and SRA accession numbers for QuadZero and AnnaLie are listed in Table 1.

**TABLE 1:** Phage accession numbers for GenBank and SRA and genome assembly results

Phage name	Genbank accession no.	SRA accession no.	Avg coverage (x)	Cluster	Genome length (bp)	GC content (%)	No. of genes
QuadZero	MZ150787	SRX12371995	1787	EA	40,140	63.6%	62
AnnaLie	MT818427	SRX12371994	842	EB	41,665	66.6%	71
